# Peptide YY_3–36_ concentration in acute- and long-term recovered anorexia nervosa

**DOI:** 10.1007/s00394-020-02210-7

**Published:** 2020-03-12

**Authors:** Friederike I. Tam, Maria Seidel, Ilka Boehm, Franziska Ritschel, Klaas Bahnsen, Ronald Biemann, Kerstin Weidner, Veit Roessner, Stefan Ehrlich

**Affiliations:** 1 Division of Psychological and Social Medicine and Developmental Neurosciences, Faculty of Medicine, Technische Universität Dresden, Fetscherstraße 74, 01307 Dresden, Germany; 2 Department of Child and Adolescent Psychiatry, Faculty of Medicine, Eating Disorder Treatment and Research Center, Technische Universität Dresden, Dresden, Germany; 3 Institute for Clinical Chemistry and Pathobiochemistry, Otto-Von-Guericke University Magdeburg, Magdeburg, Germany; 4 Department of Psychotherapy and Psychosomatic Medicine, Faculty of Medicine, Technische Universität Dresden, Dresden, Germany; 5 Department of Child and Adolescent Psychiatry, Faculty of Medicine, University Hospital C. G. Carus, Technische Universität Dresden, Dresden, Germany

**Keywords:** Peptide YY, PYY, PYY_3–36_, Anorexia nervosa, Recovered anorexia nervosa, Gut–brain axis

## Abstract

**Purpose:**

The gut–brain axis could be a possible key factor in the pathophysiology of anorexia nervosa. The neuropeptide peptide YY_3–36_, secreted by endocrine L cells of the gastrointestinal tract, is a known regulator of appetite and food intake. The objective of this study was to investigate peptide YY_3–36_ plasma concentrations at different stages of anorexia nervosa in a combined cross-sectional and longitudinal design to differentiate between effects of acute undernutrition and more enduring characteristics.

**Methods:**

We measured fasting plasma peptide YY_3–36_ concentrations in young patients with acute anorexia nervosa (*n* = 47) and long-term recovered patients (*n* = 35) cross-sectionally in comparison to healthy control participants (*n* = 58), and longitudinally over the course of inpatient treatment. Physical activity was controlled as it may modulate peptide YY secretion.

**Results:**

There was no group difference in peptide YY_3–36_ concentration among young acutely underweight anorexia nervosa patients, long-term recovered anorexia nervosa patients, and healthy control participants. Longitudinally, there was no change in peptide YY_3–36_ concentration after short-term weight rehabilitation. For acute anorexia nervosa patients at admission to treatment, there was a negative correlation between peptide YY_3–36_ concentration and body mass index.

**Conclusions:**

The current study provides additional evidence for a normal basal PYY_3–36_ concentration in AN. Future studies should study multiple appetite-regulating peptides and their complex interplay and also use research designs including a food challenge.

**Electronic supplementary material:**

The online version of this article (10.1007/s00394-020-02210-7) contains supplementary material, which is available to authorized users.

## Introduction

Anorexia nervosa (AN) is a life-threatening eating disorder typically beginning in adolescence and characterized by an intense fear of weight gain and a relentless pursuit of weight loss, mostly by self-starvation. Only less than half of AN patients fully recover from the disorder, one-third shows improvement with residual symptoms, and one fifth remains long-term chronically ill [1]. The underlying mechanisms of AN are still largely unclear and a better understanding of factors contributing to the development and progression of this enigmatic disorder would be the foundation for improving therapeutic interventions. Recently, the gut–brain axis, which describes the bidirectional communication between gut and brain via neurons, immune mediators, gut hormones, and influence of the gut microbiota [2], has been discussed as a possible key factor in the pathophysiology of AN.

Peptide YY (PYY) belongs to the neuropeptide Y family of biologically active peptides that are considered important mediators of the gut–brain axis [2]. It is a 36-amino acid peptide that is thought to be a satiety factor inhibiting food intake, gastrointestinal motility, and secretion, and may interact with the gut microbiota [2–6]. More recently, PYY has also been implicated as a possible regulator of glucose homeostasis [7]. PYY is expressed by endocrine cells of the digestive system, mostly by the endocrine L cells of the gastrointestinal tract in co-secretion with the incretin hormone glucagon-like-peptide 1 but also by endocrine cells in the pancreas and enteric neurons of the stomach [2, 7]. Its release from L cells into the blood stream is triggered by meal intake in proportion to energy intake and depending on meal composition, as well as stimulated by gastric acid secretion and a number of metabolites (for example, cholecystokinin or short chain fatty acids produced by the gut microbiota) [2, 8]. After its release, part of the parent peptide PYY_1–36_ is cleaved enzymatically by dipeptidyl peptidase 4 (DPP-4) to the main circulating form PYY_3–36_ [2, 9]. This cleavage is accompanied by a shift in the stimulated Y receptor subtypes leading to possibly divergent actions of PYY_1–36_ and PYY_3–36_ regarding energy and glucose homeostasis [2, 7, 10, 11]. Out of the two human forms of PYY, PYY_3–36_ is considered to be the one relevant to energy homeostasis [7]. The effects of PYY_3–36_ are mostly mediated through agonism at Y2 receptors on neurons both in the gastrointestinal tract and in the central nervous system [2]. Translational research implicates the arcuate nucleus of the hypothalamus and certain brain stem regions as key areas of central appetite-regulating circuits influenced by PYY_3–36_ [12]. Batterham et al. [13] found that peripherally infused PYY_3–36_ leads to a significant reduction in appetite and food intake in healthy, non-obese volunteers. Evidence from functional magnetic resonance imaging (fMRI) studies points to an influence of PYY concentration on neural activity in brain regions involved in reward processing (such as the orbito-frontal cortex), implicating that PYY may modulate the reward value of food [14, 15].

Relatively few studies have investigated PYY concentration in AN yielding heterogeneous results, as highlighted in Table 1, and only one study has investigated PYY in peripheral blood in long-term recovered AN patients. While a number of studies found a normal fasting PYY concentration in acutely underweight AN [16–20], some studies reported an elevated PYY concentration [21–25]. From a physiological perspective, one would expect the anorexigenic PYY to have low levels in this underweight and undernourished population. An elevated PYY concentration would be a maladaptive response as it may facilitate restrictive eating in AN. However, when interpreting previous studies, some confounding factors should be taken into consideration. For instance, physical exercise was shown to significantly increase the peripheral PYY concentration. Suggestive of short-term effects, Larson-Meyer et al. [26] reported elevated PYY concentrations following a 60-min run which gradually returned to baseline over the span of 120 min. Short aerobic exercise (3 × 10 min of rope skipping or cycling) also led to a brief PYY increase [27]. Similarly, Broom et al. [28] found an increase in PYY concentration after aerobic exercise (60-min run) but no difference in PYY levels among the exercise and non-exercise groups 7 h post-exercise. The majority of acutely underweight AN patients engage in excessive physical activity (reported prevalences range from 31 to 80% [29]) and it seems possible that exercise effects bias the assessment of PYY concentrations in AN. Furthermore, only a few studies assessed the biologically more relevant isoform PYY_3–36_ instead of total PYY [16, 21, 24, 30].

**Table 1:** PYY research on anorexia nervosa in humans

Author (year)	Sample size AN group (*n*)	Group comparison	Correlation with BMI	Assay	Tested PYY form (in peripheral blood)
Eddy et al. (2015) [21]	75	PYY total: AN > HC PYY_3-36_: AN > HC	All: yes (−)	RIA a RIA a	PYY total PYY_3-36_
Fernández-Aranda et al. (2016) [16]	64	AN = HC = OB	Not reported	ELISA a	PYY_3-36_
Germain et al. (2007) [17]	12	AN = HC AN < CT	Not reported	RIA c	PYY total
Germain et al. (2010) [30]	32	AN < HC	Not reported	ELISA c	PYY_3-36_
Lawson et al. (2011) [42]	16	AN > OB (trend: AN > HC)	Not reported	RIA a	PYY total
Misra et al. (2006) [23]	23	AN > HC (trend: AN T1 > AN T2)	All: yes (−)	RIA d	PYY total
Misra et al. (2008) [22]	34	AN > HC	Not reported	RIA d	PYY total
Nakahara et al. (2007) [24]	14	AN > HC AN T1 = AN T2	Not reported	RIA b	PYY_3-36_
Otto et al. (2007) [18]	16	AN = HC	No	ELISA b	PYY total
Pfluger et al. (2007) [25]	18	AN > HC AN T1 = AN T2	All: yes (−)	ELISA b	PYY total
Rigamonti et al. (2011) [43]	7 acAN 4 recAN	AN = recAN AN > OB	All: yes (−)	RIA a	PYY total
Sedlackova et al. (2012) [19]	14	HC = AN	Not reported	RIA a	PYY total
Stock et al. (2005) [20]	10	HC = AN = OB	No	RIA c	PYY total
Utz et al. (2008) [45]	12	AN only	AN: yes (−)	RIA d	PYY total

The presented group comparisons are for fasting PYY_3–36_ or total PYY concentrations only. Some of the listed studies additionally investigated PYY response to potentially stimulating factors such as food intake and some included additional patient groups

*AN* anorexia nervosa, *CT* constitutionally thin, *ELISA* enzyme-linked immunosorbent assay, *HC* healthy control participants, *OB* obese/overweight, *recAN* recovered from anorexia nervosa, *RIA* radioimmunoassay, *T1* timepoint 1 (longitudinal design), *T2* timepoint 2 (longitudinal design). Assays: RIA *a* Linco Research/Millipore Corp., MO, USA; *b* Peninsula Laboratories, CA, USA; *c* “established in-house”, *d* Phoenix Pharmaceuticals, CA, USA, *ELISA a* BioVendor Research and Diagnostic Products, Czech Republic, *b* Diagnostic Systems Laboratories Inc., TX, USA, *c* Phoenix, CA, USA

Gaining a broader understanding of the role of gut hormones such as PYY in the etiology and maintenance of AN holds the potential of identifying prognostic biomarkers and opening up new avenues for treatment. To date, no study on PYY in AN has taken physical activity into account, even though it is likely to be of great importance as a confounding factor in this patient sample. Furthermore, there is a considerably research gap regarding the persistence of possible changes after short-term and long-term weight recovery. Thus, the objective of the current study was to investigate PYY_3–36_ concentrations of patients with acutely underweight AN, before and after partial weight recovery (longitudinal sample), as well as in long-term recovered patients in comparison to healthy control participants (cross-sectional sample). The inclusion of long-term recovered patients allows the differentiation of state markers (i.e., related to acute undernutrition) from factors that might confer vulnerability towards AN. To control for possible exercise-related bias on PYY in AN, blood samples were obtained shortly after patients were admitted to an intensive treatment program which helps patients resist the drive to exercise.

## Materials and methods

### Participants

A total of 140 female subjects participated in this study: 47 underweight patients with acute AN (acAN, 12–23 years old), 35 patients long-term recovered from AN (recAN, 15–28 years old), and 58 healthy control participants (HC, 12–26 years old). For the longitudinal arm of our study, 32 acAN patients were reassessed after short-term weight rehabilitation. Participants with acAN were admitted to intensive treatment of an eating disorder program at a child and adolescent psychiatry and psychosomatic medicine department of a tertiary care university hospital. Newly admitted patients spent their first days under close supervision by specialized nursing staff and continuous heart rate monitoring at night. Therefore, we can ensure an exercise-free time period of at least 18 h before blood sample collection. Diagnosis of AN was established using the expert form of the Structured Interview for Anorexia and Bulimia Nervosa (SIAB-EX) [31] and required a body mass index (BMI) below the 10th age percentile (if younger than 15.5 years) or below 17.5 kg/m^2^ (if older than 15.5 years). RecAN participants were required to have had a diagnosis of AN in the past and, for at least 6 months before the study, (1) to have maintained a BMI above the 10th age percentile (if younger than 18 years) or above 18.5 kg/m^2^ (if older than 18 years), (2) to menstruate, and (3) to not have binged, purged, or engaged in substantial restrictive eating patterns. HC had to be of normal weight, eumenorrhoeic, and without any history of psychiatric illness. All HC were assessed with the SIAB-EX [31] and excluded if they showed any abnormal eating behavior. HC were recruited through advertisement among middle school, high school, and university students. While recAN and HC participants were only assessed once, acAN participants were assessed within 96 h of admission to intensive treatment (timepoint 1, acAN-T1) and reassessed after short-term weight rehabilitation (timepoint 2, acAN-T2; after BMI increase of at least 13%) for the longitudinal arm of this study.

Information regarding exclusion criteria and possible confounding variables, including menstrual cycle and use of contraceptive medication, was obtained from all participants (acAN, recAN, and HC) using the SIAB-EX [31], supplemented by our own semi-structured interview and medical records. Comorbid diagnoses were taken according to standard practice from medical records and confirmed by an expert clinician with over 10 years of experience. Participants of all groups were excluded if they had a history of any of the following diagnoses: organic brain syndrome, schizophrenia, substance dependence, psychosis not otherwise specified, bipolar disorder, bulimia nervosa, or binge-eating disorder. Further exclusion criteria for all participants were an IQ below 85; current substance abuse; inflammatory, neurologic, or metabolic illness; chronic medical or neurological illness that could affect appetite, eating behavior or body weight; clinically relevant anemia; pregnancy or breast feeding. Psychotropic medication within 4 weeks before the study was an additional exclusion criterion for all groups, with the exception of selective serotonin reuptake inhibitors in the acAN and the recAN group.

### Clinical measures

Additional to the evaluation with the SIAB-EX [31], eating disorder-specific psychopathology was assessed with the German version of the self-report questionnaire Eating Disorder Inventory-2 (EDI-2) [32]. Depressive symptoms were explored using the German version of the Beck Depression Inventory-II (BDI-II) [33] and general levels of psychopathology and anxiety symptoms using the revised Symptom Checklist 90 (SCL-90-R) [34]. Pre-treatment physical activity within the 3 months before inclusion into our study was assessed using the corresponding module of the SIAB-EX as previously described by Holtkamp et al. [35] and Ehrlich et al. [36]. The intelligence quotient (IQ) was estimated with short versions of the German adaptation of the Wechsler Adult Intelligence Scale (Wechsler Intelligenztest für Erwachsene) [37] or the Wechsler Intelligence Scale for Children (Hamburg-Wechsler Intelligenztest für Kinder IV) [38] for study participants aged 15 years or younger. Demographic and clinical study data were collected and managed using the secure, web-based electronic data capture tool REDCap (Research Electronic Data Capture) [39].

### Procedure

Venous blood samples were collected into vacutainer tubes between 7 and 9 a.m. after an overnight fast, for the acAN group at the first timepoint within 96 h after initiating intensive treatment. Aprotinin (Sigma-Aldrich, St. Louis, Missouri, USA) was added during blood sampling to prevent protein degradation by serine proteases. Samples were immediately centrifuged (800×*g* for 15 min) in a pre-cooled centrifuge (5 °C), aliquoted, and stored at − 80 °C. Plasma PYY_3–36_ and leptin concentrations were measured according to the manufacturer's instructions. To prevent enzymatic in-vitro degradation of PYY, DPP-4 inhibitor (Millipore S.A.S, Molsheim, France) was added before thawing of samples in a concentration of 10 µl/ml. Plasma PYY_3–36_ concentration was analyzed in duplicate using the commercially available human PYY_3–36_-specific radioimmunoassay (RIA, EMD Millipore Corporation, St. Louis, Missouri, USA) with an intra-assay coefficient of variation (CV) of 6–11%, inter-assay CV of 7–15%, and a lower limit of detection of 20 pg/ml. The employed RIA utilizes ^125^I-labeled PYY and a PYY_3–36_ antiserum to determine the PYY_3–36_ concentrations in the research samples and is specific to human PYY_3–36_, while PYY_1–36_ is not detectable. Plasma leptin concentration was measured using a commercially available enzyme-linked immunoabsorbent assay with an intra-assay CV of 4.2%, inter-assay CV of 6.7%, and a lower limit of detection of 0.2 ng/ml (ELISA, BioVendor Research and Diagnostic Products, Brno, Czech Republic) and served as a control variable.

### Statistical analysis

For all samples, PYY_3–36_ was measured twice and the mean was used for all statistical analyses. Due to deviations from normality, the non-parametric Kruskal–Wallis test was used to analyze PYY_3–36_ concentrations for the cross-sectional group comparison (acAN-T1, recAN, HC). The Wilcoxon signed-rank test was employed to analyze PYY_3–36_ concentrations longitudinally in the acAN group (acAN-T1, acAN-T2). Correlation analyses were performed using Spearman correlation and *p* values were corrected for multiple comparisons using the False Discovery Rate correction method of Benjamini and Hochberg [40]. Statistical analyses were performed using SPSS 25 (SPSS, Chicago, Illinois) and JASP for Bayesian statistics [41] with the goal to estimate the evidence in favor of the null hypothesis.

## Results

### Sample characteristics

Demographic and clinical characteristics are summarized in Table 2 for the cross-sectional sample (acAN-T1, recAN, HC) and in Table 3 for the longitudinal sample (acAN-T1, acAN-T2). As expected, patients with acAN-T1 had significantly lower BMI and BMI standard deviation scores (BMI-SDS) and higher levels of psychopathology (EDI-2 total score, BDI-II total score, SCL-R-90 global severity index). RecAN patients still had some residual psychopathology. As anticipated, leptin concentration was suppressed in the acAN-T1 group, increasing significantly after short-term weight rehabilitation (acAN-T2), while it was in the expected range for the recAN and the HC groups. There was no difference in IQ among groups. Physical activity was increased in inpatients with acAN-T1 in comparison to HC and recAN, and decreased significantly over the course of short-term weight rehabilitation (acAN-T2).

**Table 2:** Cross-sectional study sample: demographic and clinical characteristics

	*N*	acAN-T1	recAN	HC	*F*	*p*	Post hoc tests
Age (years)	47/35/58	15.8 ± 2.4	22.0 ± 3.0	18.6 ± 4.0	34.85	< 0.001	acAN < recAN acAN < HC recAN > HC
IQ	42/35/57	110.3 ± 13.7	109.3 ± 10.0	111.5 ± 9.6	0.44	0.646	–
BMI (kg/m^2^)	47/35/58	14.8 ± 1.4	20.9 ± 1.9	21.0 ± 2.7	133.04	< 0.001	acAN < recAN acAN < HC
BMI-SDS	47/35/58	− 3.2 ± 1.9	− 0.5 ± 0.6	− 0.1 ± 0.8	84.31	< 0.001	acAN < recAN acAN < HC
Minimal lifetime BMI (kg/m^2^)	47/35/57	14.3 ± 1.5	14.3 ± 1.8	19.9 ± 2.3	135.59	< 0.001	acAN < HC recAN < HC
EDI-2 (total score)	44/34/58	195.8 ± 48.8	164.0 ± 44.0	138.4 ± 26.8	26.35	< 0.001	acAN > recAN acAN > HC recAN > HC
BDI-II (total score)	47/35/58	18.8 ± 11.2	8.4 ± 8.0	4.8 ± 5.4	37.42	< 0.001	acAN > recAN acAN > HC
SCL-90-R (global severity index)	47/35/58	0.79 ± 0.65	0.44 ± 0.39	0.32 ± 0.40	12.16	< 0.001	acAN > recAN acAN > HC
Leptin (µg/l)	47/35/58	2.1 ± 3.3	9.7 ± 5.8	12.1 ± 9.1	29.16	< 0.001	acAN < recAN acAN < HC
Physical activity	45/35/57	2.4 ± 1.5	1.5 ± 1.0	1.7 ± 0.9	8.42	< 0.001	acAN > recAN acAN > HC

Mean values ± standard deviation for each variable are shown separately for each sample. Group differences were tested using ANOVA and post-hoc Scheffé

*acAN-T1* acute anorexia nervosa participants at timepoint 1 (admission), *BDI-II* Beck Depression Inventory, *BMI* body mass index, *BMI-SDS* body mass index standard deviation score, *EDI-2* Eating Disorder Inventory-2, *HC* healthy control participants, *recAN* long-term recovered anorexia nervosa participants, *SCL-90-R* Symptom Checklist-90-Revised

In the acAN group, the mean age of illness onset was 13.5 ± 1.9 years and the mean duration of illness in the acAN group was 17.9 ± 25.0 months. In the acAN group, 42 (89.4%) were of the restrictive subtype and 5 (10.6%) were of the binge/purge subtype. The mean duration since weight normalization in the recAN group was 50.3 ± 34.1 months. In the recAN group, 27 (77.1%) used to be of the restrictive AN subtype and 8 (22.9%) used to be of the binge/purge AN subtype. Of the acAN participants at T1, 5/47 had psychiatric comorbidities: 3/47 had a depressive disorder, 1/47 reported selective mutism, and 1/47 had a major depressive disorder, a generalized anxiety disorder and social phobia, an obsessive–compulsive disorder, and a combined personality disorder. Of the recAN participants, 8/35 had psychiatric comorbidities: 7/35 had a depressive disorder, and 1/35 had an obsessive–compulsive disorder. None of the acAN-T1, recAN, or HC participants were on psychoactive medication

**Table 3:** Longitudinal sample: demographic and clinical characteristics

	*N*	acAN-T1	acAN-T2	*t*	*p*
Age (years)	32/32	15.3 ± 2.5	15.6 ± 2.5	− 11.23	< 0.001
BMI (kg/m^2^)	32/32	14.8 ± 1.1	18.6 ± 1.1	− 20.01	< 0.001
BMI-SDS	32/32	− 2.8 ± 1.1	− 0.7 ± 0.7	− 16.9	< 0.001
EDI-2 (total score)	28/28	190.5 ± 43.4	183.1 ± 43.2	1.24	0.225
BDI-II (total score)	30/30	17.9 ± 10.3	9.7 ± 8.5	5.10	< 0.001
SCL-90-R (global severity index)	31/31	0.72 ± 0.56	0.42 ± 0.36	4.53	< 0.001
Leptin (µg/l)	32/32	2.6 ± 3.7	12.7 ± 7.1	− 7.17	< 0.001
Physical activity	31/31	2.4 ± 1.4	1.6 ± 1.2	2.88	0.007

Mean values ± standard deviation for each variable are shown separately for each timepoint. Differences between timepoints were tested using paired-sample *t* tests

*acAN* acute anorexia nervosa participants, *BDI-II* Beck Depression Inventory, *BMI* body mass index, *EDI-2* Eating Disorder Inventory-2, *HC* healthy control participants, *recAN* long-term recovered anorexia nervosa participants, *SCL-90-R* Symptom Checklist-90-Revised, *T1* timepoint 1 (at admission), *T2* timepoint 2 (after short-term weight rehabilitation). Regarding psychiatric comorbidities, 2/32 acAN participants in the longitudinal sample had a depressive disorder. None of the acAN participants in the longitudinal sample were on psychoactive medication

### PYY_3–36_ concentration

**Fig. 1:**
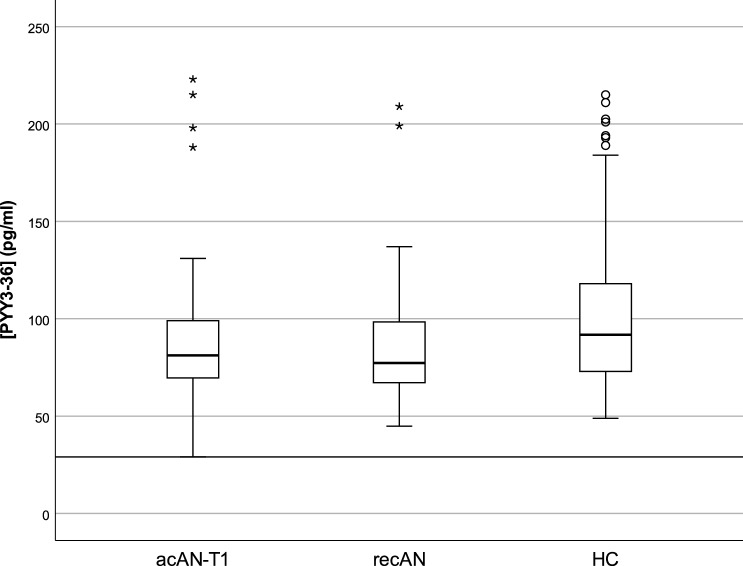
PYY_3–36_ concentrations in the cross-sectional sample. *acAN-T1* acute anorexia nervosa participants at timepoint 1 (admission), *recAN* long-term recovered anorexia nervosa participants; HC, healthy control participants. Boxplots showing the median, upper and lower quartiles, mild outliers (depicted as circles, values deviating more than 1.5 times the interquartile range from the upper or lower quartile) and extreme outliers (depicted as asterisks, values deviating more than 3 times the interquartile range from the upper or lower quartile), and the lower measurement limit indicated as a horizontal line. PYY_3-36_ concentrations: acAN-T1: median (Mdn) = 81.2 pg/ml, interquartile range (IQR) = 30.5, recAN: Mdn = 77.3 pg/ml, IQR = 32.9, HC: Mdn = 91.8 pg/ml, IQR = 46.2. 2/47 acAN-T1 participants, but none of the recAN or the HC participants had PYY_3-36_ concentrations below the lower measurement limit (29 pg/ml). The figure was created with SPSS 25 (SPSS, Chicago, Illinois)

**Fig. 2:**
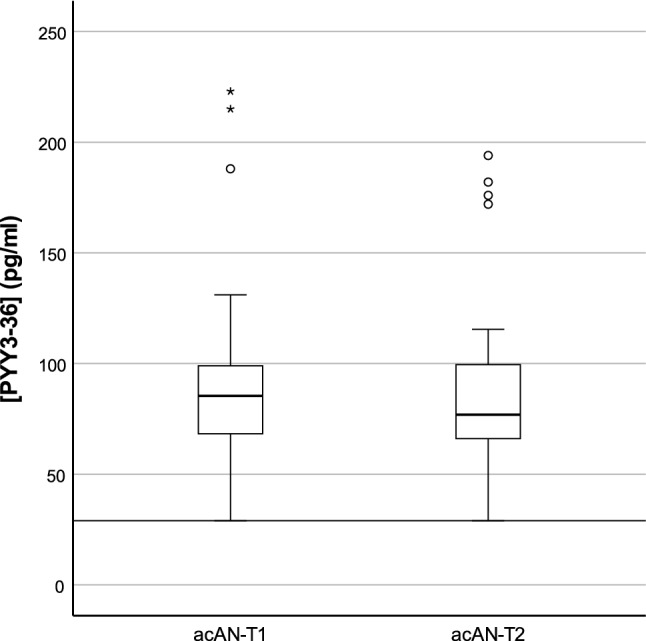
*PYY*_*3*–*36*_ concentrations in the longitudinal sample. *acAN-T1* acute anorexia nervosa participants at timepoint 1 (admission), *acAN-T2* acute anorexia nervosa participants at timepoint 2 (after short-term weight rehabilitation). Boxplots showing the median, upper and lower quartiles, mild outliers (depicted as circles, values deviating more than 1.5 times the interquartile range from the upper or lower quartile), and extreme outliers (depicted as asterisks, values deviating more than 3 times the interquartile range from the upper or lower quartile), and the lower measurement limit indicated as a horizontal line. PYY_3–36_ concentrations: acAN-T1: median (Mdn) = 85.4 pg/ml, interquartile range (IQR) = 31.1, acAN-T2: Mdn = 76.9 pg/ml, IQR = 34.9. One participant had PYY_3–36_ concentrations below the lower measurement limit (29 pg/ml) for both timepoints. The figure was created with SPSS 25 (SPSS, Chicago, Illinois)

There was no significant difference in PYY_3–36_ concentration among the acAN-T1, recAN, and HC groups, *H*(2) = 4.04, *p* = 0.133 (Fig. 1). Bayesian statistics provided additional support for the null hypothesis (Supplemental Material 1.1). In our longitudinal sample of acAN participants, the PYY_3–36_ concentration did not change over the course of short-term weight rehabilitation, *T* = 178, *p* = 0.170, *r* = − 0.17 (Fig. 2).

Associations of PYY_3–36_ with BMI, BMI-SDS, age, leptin concentration, pre-treatment physical activity (for the acAN group), duration of recovery (for the recAN group), and measures of psychopathology (BDI-II total score, EDI-2 total score, SCL-90-R global severity index) were tested for each group using Spearman correlations. For the acAN-T1 group, there was a negative correlation between PYY_3–36_ and BMI (*rs* = -0.45, *p* = 0.001, Benjamini–Hochberg corrected *p* = 0.009), but no significant correlation of PYY_3–36_ with any of the other parameters including age-adjusted BMI-SDS and pre-treatment physical activity. There were no significant associations between PYY_3–36_ and demographic and clinical variables for the acAN group at T2, the recAN, or the HC groups. An exploratory analysis to test a possible influence of the duration of realimentation and BMI change over the course of treatment on PYY_3–36_ concentrations in the longitudinal acAN study population revealed no significant influence of these factors (Supplemental Material 1.2).

## Discussion

In the present study, we investigated plasma PYY_3–36_ concentrations in patients with acute AN and individuals after long-term recovery from AN cross-sectionally in comparison to healthy control participants, and longitudinally over the course of inpatient treatment. To specifically measure the isoform most relevant to energy homeostasis [7], we chose a PYY_3–36_-specific RIA. There was no group difference in PYY_3–36_ concentrations. Our longitudinal analysis in acute AN patients also yielded no change in PYY_3–36_ concentration after short-term weight rehabilitation. At admission to treatment, we found a negative correlation between PYY_3–36_ concentration and BMI.

The results of the present study are in line with a number of studies reporting a normal PYY concentration in acute AN [16–20]. However, others have found elevated PYY levels in acute AN [21–25] and one study, measuring circadian levels of PYY, reported lower concentrations in AN in comparison to a healthy control group [30]. This heterogeneity of results may be explained at least in part by the use of assays targeting total PYY (sum of PYY_1–36_ and PYY_3–36_) instead of PYY_3–36_ [17–20, 22, 23, 42, 43]. The importance of differentiating between PYY_1–36_ and PYY_3–36_ is further underlined by a recent study demonstrating different physiological effects of the two isoforms [10]. Last but not least, physical activity may be a possible confounding factor especially relevant for this study population as patients in the acute stage of the disorder often engage in excessive exercise. Multiple studies have reported an exercise-induced increase of PYY concentrations in healthy subjects [26–28]. The duration of the exercise-related effect on PYY concentration appears to depend on the type and intensity of the physical activity, but does not seem to exceed a few hours. Given the intensive treatment setting at the time of venipuncture in our study, we were able to minimize acute effects of excessive physical activity on PYY. Our finding of a lack of correlation between the PYY concentration and pre-treatment physical activity supports this assumption.

Two longitudinal studies, which reported no significant change in PYY concentrations after weight gain, are in accordance with our findings [24, 25]. To our knowledge, there are only two studies investigating PYY in long-term recovered AN patients, with study designs that significantly limit comparability to our results [43, 44]. In line with our findings, Rigamonti et al. [43] did not report a significant difference in total PYY concentrations among acAN and recAN, but the sample size was very small (acAN *n* = 7, recAN *n* = 4) and no healthy control group was included in the study. Gendall et al. [44] also reported normal total PYY levels for recAN patients, but PYY was measured in cerebrospinal fluid instead of blood.

Our finding of a negative correlation between PYY concentration and BMI in the acute AN group before treatment is consistent with the results of a number of studies [21, 23, 25, 43, 45]. While our result should be interpreted cautiously as it was not confirmed for age-adjusted BMI-SDS, it is possible that a higher PYY concentration contributes to the severity of the disorder by reducing appetite and food intake. On the other hand, a low PYY concentration might be a protective factor against severe undernutrition in a subgroup of patients as it may counteract self-starvation by increasing appetite and food intake. In this context, Misra et al. [23] remarked a strong negative association between fasting PYY levels and fat intake in girls with AN.

When considering our findings, some limitations have to be taken into account. First, the sample size may not have been sufficient to draw definite conclusions regarding basal PYY_3–36_ concentrations in AN. Second, taking into account the complexity of the function of PYY as a regulator of energy homeostasis, measurement of fasting concentrations is only one approach to investigate its role in the etiology and progression of AN and future studies in acute AN should also target the PYY_3–36_ response to standard meals. To date, only four studies investigated PYY in response to meal intake in AN with mixed results [18–20, 24] and only one of these measured PYY_3–36_ instead of total PYY [24]. Third, because of the possibly divergent actions of the two human isoforms PYY_1–36_ and PYY_3–36_, we chose an assay that targets only the isoform PYY_3–36_, which is assumed to be more relevant for energy homeostasis. Thus, our results allow no conclusions regarding the physiological effects of PYY_1–36_ and measurement of both, total PYY (the sum of PYY_1–36_ and PYY_3–36_) and PYY_3–36_, yielding their ratio (i.e., total PYY/PYY_3–36_ or PYY_1–36_/PYY_3–36_), would allow for a more comprehensive picture of the peptide’s metabolism in AN.

In conclusion, the current study provides additional evidence for a normal basal PYY_3–36_ concentration in AN. Future studies should further measure both PYY_1–36_ and PYY_3–36_, explore their levels in response to food intake in AN, and take physical activity as a possible confounding factor into consideration. Another promising approach would be to simultaneously study multiple appetite-regulating peptides and their complex interplay.

## Electronic supplementary material

Below is the link to the electronic supplementary material.

Supplementary file1 (PDF 384 kb)
