# Supplementary material for: Peptide YY3–36 concentration in acute- and long-term recovered anorexia nervosa
Source: Eur J Nutr. 2020 Mar 12;59(8):3791–9. doi: 10.1007/s00394-020-02210-7 (PMC7669786; doi:10.1007/s00394-020-02210-7)
Supplement: Supplementary file 1 — Supplementary file1 (PDF 384 kb) [file 394_2020_2210_MOESM1_ESM.pdf]

Supplementary Material for Peptide YY_{3-36} concentration in acute and long-term recovered anorexia nervosa

Journal: European Journal of Nutrition

Authors:

Friederike I. Tam[a,b]

Maria Seidel[a,b]

Ilka Boehm[a,b]

Franziska Ritschel[a]

Klaas Bahnsen[a]

Ronald Biemann[c]

Kerstin Weidner[d]

Veit Roessner[e]

Stefan Ehrlich[a,b]

[a] Division of Psychological and Social Medicine and Developmental Neurosciences, Faculty of Medicine, Technische Universität Dresden, Dresden, Germany.

[b] Department of Child and Adolescent Psychiatry, Faculty of Medicine, Eating Disorder Treatment and Research Center, Technische Universität Dresden, Dresden, Germany.

[c] Institute for Clinical Chemistry and Pathobiochemistry, Otto-von-Guericke University Magdeburg, Magdeburg, Germany.

[d] Department of Psychotherapy and Psychosomatic Medicine, Faculty of Medicine, Technische Universität Dresden, Dresden, Germany.

[e] Department of Child and Adolescent Psychiatry, Faculty of Medicine, University Hospital C. G. Carus, Technische Universität Dresden, Dresden, Germany.

Corresponding author: Stefan Ehrlich, M.D., Division of Psychological and Social Medicine and Developmental Neurosciences, Faculty of Medicine, Technische Universität Dresden, Fetscherstraße 74, 01307 Dresden, Germany, phone number: +49 351 458-5214, email: transden.lab@uniklinikum-dresden.de

SM 1 Supplemental Results

SM 1.1 Control analyses: Bayesian statistics

We employed Bayesian statistics conducted with JASP [1] to verify our results. One advantage of these tests is the possibility to test which hypothesis is best supported by the data and to specifically also test the evidence in favor of the null hypothesis (H_0).

From the results of prior studies on PYY and $PYY_{3\text{-}36}$ in AN, it is possible to make an informed assumption about the pair-wise comparison between acAN and HC. To determine a likely effect size for the comparison between acAN and HC, we aimed to include all studies about total PYY or $PYY_{3\text{-}36}$ in AN with a representative sample size of n>20 [2–6]. However, Germain et al. [5] was not included since the statistical parameters necessary to determine an effect size were not reported, and Misra et al., 2006 [6] was not included as Misra et al., 2008 [4] used data from the same subjects in their analyses. Thus, we used the results of the remaining three studies [2–4] to calculate the Cohen's *d* statistic for the pair-wise comparison between AN and HC participants according to the following formula:

$$d = \frac{M_1 - M_2}{SD_{pooled}}$$

where M_1 and M_2 are the means of the AN group and the HC group and SD_{pooled} is the pooled standard deviation, calculated according to the following formula (SD_1 and SD_2 are the standard deviations of the AN group and the HC group):

$$SD_{pooled} = \sqrt{\frac{(SD_1^2 + SD_2^2)}{2}}$$

The calculated effect sizes were d=10.98 for Eddy et al. [2] who measured $PYY_{3\text{-}36}$ in 75 AN and 22 HC, d=0.33 for Fernández-Aranda et al. [3] who measured total PYY in 64 AN and 80 young HC, and d=1.60 for Misra et al. [4] who measured total PYY in 34 AN and 33 HC. The mean effect size for these three studies was d=4.30, which provides strong a priori evidence for the alternative hypothesis PYY: AN>HC.

Therefore, we conducted a Bayesian two-sample t test to test whether the data best supported the alternative hypothesis $PYY_{3\text{-}36}$: acAN>HC (H_+) or H_0. The Bayes factor BF_{0+} (H_0/H_+) suggested that the data were 11.6:1 in favor of the null hypothesis (strong evidence, Figure S1).

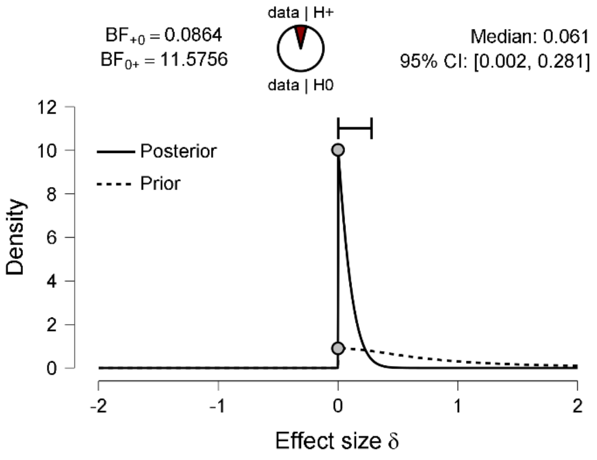

Figure S1. *Output of the Bayesian two-sample t-test performed with JASP* [1]. The probability wheel visualizes the evidence that the data provide for the null hypothesis H_0 and the one-sided alternative hypothesis H_+ (PYY_{3-36}: acAN > HC). The population effect size δ was assigned a Cauchy prior distribution with a prior width $r = \frac{1}{\sqrt{2}}$, truncated to allow only positive effect size values.

For the comparison between recAN and HC, there were no previous studies of PYY in blood. So there were two possible assumptions: the increase of PYY in acute AN observed in previous studies could a) (partially) persist or b) completely normalize with weight recovery. Therefore, we conducted a Bayesian two-sample t test to test whether the data best supported the alternative hypothesis PYY_{3-36}: recAN > HC (H_+) or H_0. The Bayes factor BF_{0+} (H_0/H_+) suggested that the data were 12.2:1 in favor of the null hypothesis (strong evidence, Figure S2).

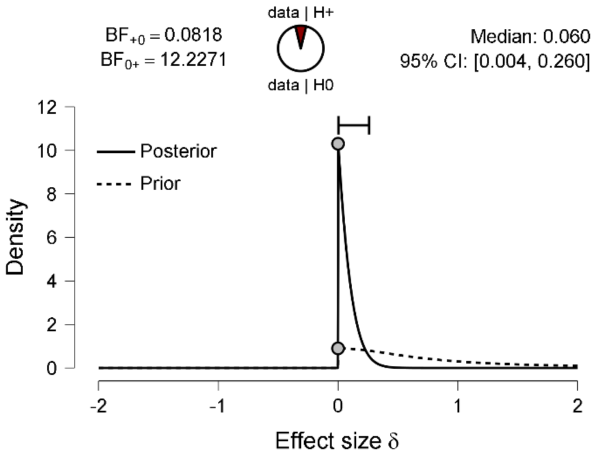

Figure S2. *Output of the Bayesian two-sample t-test performed with JASP* [1]. The probability wheel visualizes the evidence that the data provide for the null hypothesis H_0 and the one-sided alternative hypothesis H_+ (PYY_{3-36}: recAN > HC). The population effect size δ was assigned a Cauchy prior distribution with a prior width $r = \frac{1}{\sqrt{2}}$, truncated to allow only positive effect size values.

SM 1.2 Control analyses: Effects of duration of realimentation and BMI change

We conducted an exploratory analysis to test a possible influence of the duration of realimentation and BMI change from timepoint 1 (T1, admission to treatment) to timepoint 2 (T2, after short-term weight restoration) on PYY_{3-36} concentrations in the longitudinal AN study population (see Table S1 for statistical parameters). The median BMI change from T1 to T2 was 24.3% (interquartile range (IQR)=12.2, minimum=13.7%, maximum=47.9%). The median duration of realimentation from T1 to T2 was 84.5 days (IQR=27.8, minimum=51 days, maximum=256 days).

We divided the longitudinal study population into two subgroups: one subgroup with a duration of realimentation below the group median of 84.5 days and one subgroup with a duration of realimentation greater than or equal to the median. In the subgroup with shorter duration of realimentation, a Wilcoxon signed-rank test indicated no significant difference in PYY_{3-36} concentration between T1 and T2. Similarly, in the subgroup with longer duration of realimentation, there was no significant difference in PYY_{3-36} concentration between T1 and T2. Dividing the longitudinal study population into two subgroups on the basis of BMI change yielded a similar result. In the subgroup with a BMI change below the median of 24.3%, there was no significant difference in PYY_{3-36} concentration between T1 and T2. Similarly, in the subgroup with a BMI change greater or equal to 24.3%, there was no significant difference in PYY_{3-36} concentration between T1 and T2.

As an alternative approach, a repeated measures general linear model showed no significant difference between PYY_{3-36} concentrations at T1 and T2 after controlling for duration of realimentation and percentage of BMI change, $F(1,29)=0.26$, $p=0.616$.

Furthermore, we tested if the correlation between PYY_{3-36} concentration and BMI-SDS in the acAN group at T2 was influenced by duration of realimentation or BMI change from T1 to T2 (see Table S2 for statistical parameters). In the subgroup with shorter duration of realimentation, there was no significant relationship between PYY_{3-36} concentration at T2 and BMI-SDS at T2. Similarly, in the subgroup with longer duration of realimentation, there was no significant relationship between PYY_{3-36} concentration at T2 and BMI-SDS at T2. In the subgroup with a BMI change below the median of 24.3%, there was no significant relationship between PYY3-36 concentration at T2 and BMI-SDS at T2. Similarly, in the subgroup with a BMI change greater or equal to 24.3%, there was no significant relationship between PYY_{3-36} concentration at T2 and BMI-SDS at T2.

Table S1. *Wilcoxon signed-rank test comparing $PYY_{3\text{-}36}$ concentrations in different subgroups between acAN-T1 and acAN-T2.*

	n	Median	IQR	T	z	p
$PYY_{3\text{-}36}$ (pg/ml)						
Short duration of realimentation						
acAN-T1	16	82.8	26.4	59	-0.47	0.642
acAN-T2	16	75.5	28.4			
$PYY_{3\text{-}36}$ (pg/ml)						
Long duration of realimentation						
acAN-T1	16	95.2	61.2	34	-1.48	0.140
acAN-T2	16	77.7	86.3			
$PYY_{3\text{-}36}$ (pg/ml)						
Low BMI Change						
acAN-T1	16	79.5	25.0	54	-0.34	0.733
acAN-T2	16	76.9	33.7			
$PYY_{3\text{-}36}$ (pg/ml)						
High BMI Change						
acAN-T1	16	97.6	42.4	40	-1.45	0.148
acAN-T2	16	78.4	37.6			

Abbreviatios: acAN-T1, acute anorexia nervosa participants at timepoint 1 (admission); acAN-T2, acute anorexia nervosa participants at timepoint 2 (after short-term weight rehabilitation); BMI, body mass index; IQR, interquartile range. Short duration of realimentation was defined as below the group median of 84.5 days, long duration of realimentation was defined as equal or greater than 84.5 days. Low BMI change was defined as below the group median of 24.3%, high BMI change was defined as equal or greater than 24.3%.

Table S2. *Correlations of $PYY_{3\text{-}36}$ concentrations with BMI-SDS at acAN-T2 in different subgroups.*

	BMI-SDS
$PYY_{3\text{-}36}$ (pg/ml) acAN-T2<br>Short duration of realimentation	r_s=-0.194<br>p=0.471
$PYY_{3\text{-}36}$ (pg/ml) acAN-T2<br>Long duration of realimentation	r_s=0.153<br>p=0.572
$PYY_{3\text{-}36}$ (pg/ml) acAN-T2<br>Low BMI Change	r_s=-0.006<br>p=0.983
$PYY_{3\text{-}36}$ (pg/ml) acAN-T2<br>High BMI Change	r_s=-0.041<br>p=0.880

Spearmann correlation coefficients and p values were reported. There were no statistically significant correlations. Abbreviations: acAN-T1, acute anorexia nervosa participants at timepoint 1 (admission); acAN-T2, acute anorexia nervosa participants at timepoint 2 (after short-term weight rehabilitation); BMI-SDS, body mass index standard deviation score; IQR, interquartile range. Short duration of realimentation was defined as below the group median of 84.5 days, long duration of realimentation was defined as equal or greater than 84.5 days. Low BMI change was defined as below the group median of 24.3%, high BMI change was defined as equal or greater than 24.3%.

References cited here:

1. JASP Team (2019) JASP (Version 0.11.1) [Computer software]
2. Eddy KT, Lawson EA, Meade C, Meenaghan E, Horton SE, Misra M, Klibanski A, Miller KK (2015) Appetite Regulatory Hormones in Women With Anorexia Nervosa: Binge-Eating/Purging Versus Restricting Type. J Clin Psychiatry 76:19–24. https://doi.org/10.4088/JCP.13m08753
3. Fernández-Aranda F, Agüera Z, Fernández-García JC, Garrido-Sanchez L, Alcaide-Torres J, Tinahones FJ, Giner-Bartolomé C, Baños RM, Botella C, Cebolla A, Torre R de la, Fernández-Real JM, Ortega FJ, Frühbeck G, Gómez-Ambrosi J, Granero R, Islam MA, Jiménez-Murcia S, Tárrega S, Menchón JM, Fagundo AB, Sancho C, Estivill X, Treasure J, Casanueva FF (2016) Smell–taste dysfunctions in extreme weight/eating conditions: analysis of hormonal and psychological interactions. Endocrine 51:256–267. https://doi.org/10.1007/s12020-015-0684-9
4. Misra M, Prabhakaran R, Miller KK, Goldstein MA, Mickley D, Clauss L, Lockhart P, Cord J, Herzog DB, Katzman DK, Klibanski A (2008) Prognostic Indicators of Changes in Bone Density Measures in Adolescent Girls with Anorexia Nervosa-II. J Clin Endocrinol Metab 93:1292–1297. https://doi.org/10.1210/jc.2007-2419
5. Germain N, Galusca B, Grouselle D, Frere D, Billard S, Epelbaum J, Estour B (2010) Ghrelin and Obestatin Circadian Levels Differentiate Bingeing-Purging from Restrictive Anorexia Nervosa. J Clin Endocrinol Metab 95:3057–3062. https://doi.org/10.1210/jc.2009-2196.
6. Misra M, Miller KK, Tsai P, Gallagher K, Lin A, Lee N, Herzog DB, Klibanski A (2006) Elevated peptide YY levels in adolescent girls with anorexia nervosa. J Clin Endocrinol Metab 91:1027–1033. https://doi.org/10.1210/jc.2005-1878
